# Ovarian volume, not follicle count, is independently associated with androgens in patients with polycystic ovary syndrome

**DOI:** 10.1186/s12902-022-01224-y

**Published:** 2022-12-01

**Authors:** Asieh Mansour, Amir Pejman Hashemi Taheri, Behnaz Moradi, Mohammad Reza Mohajeri-Tehrani, Mostafa Qorbani, Sahar Ghorbani Pashakolaee, Milad Sanginabadi, Sayed Mahmoud Sajjadi-Jazi

**Affiliations:** 1 Endocrinology and Metabolism Research Center, Endocrinology and Metabolism Clinical Sciences Institute, Tehran University of Medical Sciences, Tehran, Iran; 2 Radiology department, Shariati hospital, Tehran University of Medical Sciences, Tehran, Iran; 3 Department of Radiology, Tehran University of Medical Sciences, Tehran, Iran; 4 Non-communicable Diseases Research Center, Alborz University of Medical Sciences, Karaj, Iran; 5 Chronic Diseases Research Center, Endocrinology and Metabolism Population Sciences Institute, Endocrinology and Metabolism Research Institute, Tehran University of Medical Sciences, Tehran, Iran; 6 Cell Therapy and Regenerative Medicine Research Center, Endocrinology and Metabolism Molecular-Cellular Sciences Institute, Tehran University of Medical Sciences, Tehran, Iran

**Keywords:** Ovarian volume, Follicle count, Androgen, Polycystic ovary syndrome, PCOS

## Abstract

**Background:**

Polycystic ovary syndrome (PCOS) is diagnosed based on chronic anovulation, androgen excess (clinical and/or biochemical), and polycystic ovaries in ultrasound. The aim of the present study was to evaluate which parameters in the transvaginal ultrasound (TVUS) of ovaries could be better associated with concurrent hormonal imbalance in the women with PCOS.

**Methods:**

Using a cross-sectional design, this study focused on 61 subjects (18–40 years) with PCOS. Patients were recruited at three academic hospitals during the 2017–2019 period. PCOS was defined according to the Rotterdam criteria. The association of ovarian morphology with hormonal and metabolic feature was investigated using linear regression models, adjusted for a set of possible confounding variables including age, mensuration status and body mass index (BMI).

**Results:**

The mean volume of both ovaries was positively associated with the total testosterone level (β = 0.025, *P* value < 0.001), free androgen index (β = 0.041, *P* value < 0.001) and luteinizing hormone/follicle stimulating hormone (LH/FSH) ratio (β = 0.032, *P* value = 0.004), even after adjustments made for age, mensuration status and BMI (fully-adjusted model). In contrast, in the fully-adjusted model, antral follicle count (AFC), follicle number per ovary (FNPO), ovarian area, stromal area, and ratio of stromal area to ovarian area (S/A) were not associated with androgen levels and LH/FSH ratio. In addition, after full adjustments, ovarian volume, AFC, FNPO, ovarian area, stromal area and S/A were not associated with insulin resistance, which was estimated by the homeostasis model assessment of insulin resistance (HOMA-IR).

**Conclusion:**

Increased ovarian volume is, thus, highly predictive of hyperandrogenemia and high LH/FSH ratio in PCOS patients.

## Introduction

Polycystic ovary syndrome (PCOS) has become the common endocrine and metabolic disorder of women of reproductive age worldwide, with an estimated prevalence of 6–15% [1]. It is widely believed that elevated androgen levels have a negative impact on follicular development, thus with the potential to negatively affecting the pregnancy rate [2]. Nevertheless, there are still many limitations in assessing androgen levels; it is not clear which androgens, when and how often should be measured [3]. There are also no clear cut-off levels for biochemical hyperandrogenism in women [3]; further, serum androgen levels are influenced by metabolic state such as the degree of adiposity [4, 5]. Additionally, different laboratory methods are used to determine free and total testosterone concentrations as a marker of androgens status. Therefore, accurate evaluation and interpretation of androgen hormones in women can be challenging, calling for more investigation [1, 6]. As a result, some investigators have proposed surrogate markers to increase the accuracy of assessing androgen levels in women with PCOS. Thus, finding a non-invasive measure to identify androgen excess, particularly in the patients with PCOS, is of paramount importance to detect which women with PCOS are at a greater risk and needs treatment. Given the advancement of ultrasound technology, ovarian ultrasonography is proposed in the clinical evaluation of androgen excess [6]. Moreover, Rotterdam consensus (2003) and Androgen Excess & PCOS Society (2006) have included ovarian ultrasound in the diagnosis of PCOS [7]. The association between the morphology of ovaries and the laboratory parameters of PCOS has become the object of some recent studies; however, it is uncertain whether polycystic ovarian morphology (PCOM) predicts the degree of hyperandrogenemia or metabolic dysfunction in PCOS [8–10]. In addition, it is not well known which features of PCOM inform the severity or risk of hormonal and metabolic abnormality in PCOS. The aim of this study was, therefore, to evaluate whether ovarian characteristics of PCOS, as reflected by PCOM, could be associated with hyperandrogenemia and metabolic dysfunction (e.g. insulin resistance) in women with PCOS. The intention was to identify whether the association between features of PCOM and biochemical variables was independent of other parameters known to influence insulin resistance, such as age and body mass index (BMI).

## Methods

### Subjects

The present study involved the secondary analysis of pooled data from two separate studies for which protocols were available at irct.ir (IRCT2017061917139N2 and IRCT20140406017139N3). Studies were prospectively conducted at three academic hospitals in Tehran, Iran, from August, 2017 to April, 2019. Protocols were approved by the Ethics Review Board at the Tehran University of Medical Sciences. Informed consent was obtained before study procedures were initiated.

Based on the 2003 Rotterdam criteria, the diagnosis of PCOS was made when at least two of the following three findings were present: oligo/amenorrhea, clinical (excess hair growth, virilization, alopecia, or acne) or chemical (total testosterone > 0.7 ng/mL) hyperandrogenism and PCOM according to the ultrasound exam [11].

**Fig. 1:**
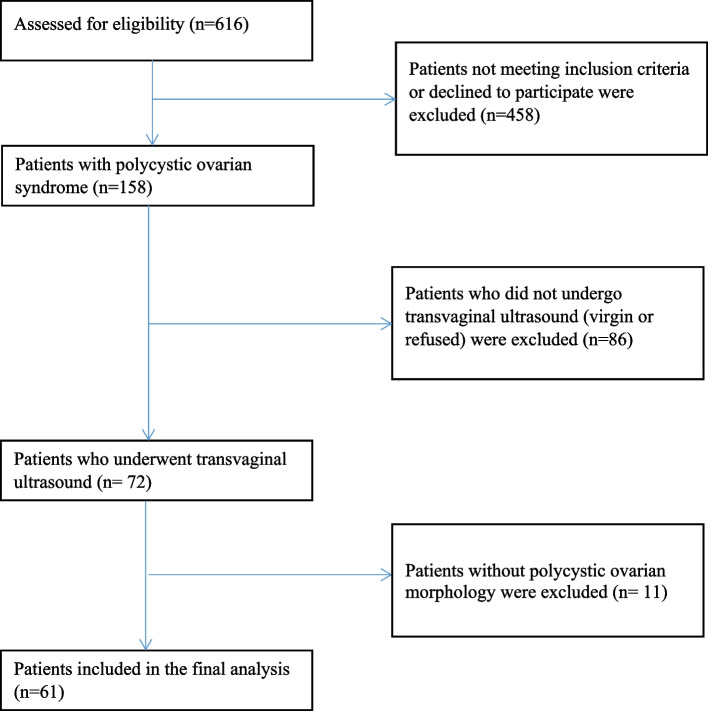
Recruitment flow of the study participants

In order to detect the association between testosterone and ovarian volume or PCOM, the sample size was estimated at 65 patients, using an alpha of 0.05 with 80% power (β = 0.2). Subjects had to be aged 18–40 years at the time of screening. None of the subjects were pregnant or had breastfeeding, diabetes, Cushing’s disease, acromegaly, ovarian insufficiency, history of ovarian surgery or any condition mimicking the PCOS (i.e., hyperprolactinemia, thyroid dysfunction or non-classical congenital adrenal hyperplasia); also, they had received no hormonal therapy (such as birth control pills), insulin sensitizers, and/or other drugs that could affect ovarian morphology in the past one month. Women who were virgin or those who declined to undergo transvaginal ultrasound (TVUS) were excluded from study. Of the 616 patients presented for evaluation, finally 61 patients with PCOS were eligible for inclusion in this study (Fig. 1).

### Clinical evaluation

All patients underwent a complete history and physical examination. The following parameters were recorded: height, weight, age, degree of ovulatory dysfunction, presence of acne and hirsutism score. Ovulatory dysfunction was then defined as oligomenorrhea (intermenstrual interval > 35 days) or amenorrhea (no menses for 3 to 6 months or longer) [12]. Hirsutism and acne were evaluated using the Ferriman-Gallwey method [13] and acne score [14]. Hyperandrogenemia was then defined as the total testosterone > 0.7 ng/mL [15].

According to anthropometric measurements, the waist was measured at the narrowest part of the torso between the costal margin and the iliac crest. In addition, each patient was weighed and her height was measured. BMI was calculated as weight divided by the square of height (kg/m^2^). Systolic and diastolic blood pressures were measured by a standard sphygmomanometer after 5 min of rest in the seated position.

### Biochemical analysis

Blood samples for the analyses of fasting blood glucose (FBS), total testosterone, sex hormone binding globulin (SHBG) and insulin were drawn in the morning after an overnight fast. Serum total testosterone, luteinizing hormone (LH), follicle stimulating hormone (FSH), prolactin, insulin, and thyroid stimulating hormone (TSH) levels were measured by using ELISA kit (Monobind Inc. Lake Forest, California, USA). SHBG was then analyzed by ELISA kits (Demeditec, Germany). Free androgen index (FAI) was calculated using the equation FAI = total testosterone (ng/mL) × 3.47/SHBG (nmol/L). Homeostasis model assessment for insulin resistance (HOMA-IR: FBS (mg/dL) × fasting insulin (µIu/mL)/405) was then used to assess insulin resistance. Prolactin and TSH were measured to rule out hyperprolactinemia and thyroid dysfunction.

### Ultrasonography measurements

Participants were evaluated by TVUS by one of two specialist ultrasonographers, using a 5-9 MHz transvaginal probe (Sonoline G40; Siemens Medical Solutions, USA, Inc.). Participants with regular menstrual cycles were evaluated in the early follicular phase between days 2 to 7 of their menstrual period; women with cycle irregularity were evaluated at random (independently of cycle day). TVUS data from both ovaries were recorded for each participant. To prevent the overestimation of the ovarian volume, if there was a dominant follicle (> 10 mm) or corpus luteum cyst in one ovary, data were reported only for the opposite one. However, if both ovaries had a dominant follicle, TVUS was repeated in the next menstrual cycle. PCOM was defined as follicle number > 25 2 to 9 mm in diameter in at least one ovary and/or ovarian volume > 10 cm^3^.

The ultrasound images were evaluated for the ovarian volume, ovarian area, stromal area, and ratio of stromal area to ovarian area (S/A) in the largest single cross-sectional view of each ovary. Ovarian area was calculated using the equation (π/4 × (transverse diameter) × (longitudinal diameter)); also, ovarian volume calculated according to the formula: π/6 × (transverse diameter) × (anteroposterior diameter) × (longitudinal diameter). The stromal area was measured by outlining the peripheral profile of stroma, taking care to avoid antral follicles represented by anechoic structures in the ovary. S/A was calculated by dividing the peripheral profile of the stroma by the peripheral profile of the ovary. The mean value of the left and right ovary was used for analysis in the ovarian area, stromal area, and S/A.

The number and diameter of all antral follicles were obtained for each ovary using the grid-system approach [16]. The antral follicle count (AFC) was then defined as the sum of the right and left of all visible follicles measured to be 2–9 mm in diameter. The follicle number per ovary (FNPO) was defined as the mean number of 2–9 mm follicles in the left and right ovary. For further analysis, FNPO was also categorized according to different follicle sizes (2–5 mm and 6–9 mm).

### Statistical analysis

Data was analyzed using SPSS Statistics for Windows, Version 16.0 (SPSS Inc., Chicago, IL, USA). Quantitative variables are presented as the mean ± standard deviation (SD). Non-normally distributed variables (FAI, LH/FSH ratio and HOMA-IR) were log transformed. Association of ovarian morphology with hormonal (testosterone, FAI and LH/FSH ratio), and metabolic features (HOMA-IR) was investigated using linear regression with the following covariates: age (model 2), age and mensuration status (model 3), age, mensuration status and BMI (model 4 or fully-adjusted model). *P* values < 0.05 were considered to be significant.

## Results

Clinical, biochemical and ultrasonographic characteristics of the patients with PCOS are reported in Table 1. The mean age of the participants was 29.92 ± 5.3 years. Fifty-two out of 61 patients had oligomenorrhea or amenorrhea, whereas the remaining 9 participants had regular menstruation. Based on the inclusion criteria, all participants had PCOM in ultrasound; among them, 41% had unilateral PCOM and 59% had bilateral PCOM. The proportions of patients with hyperandrogenemia and hyperandrogenism (clinical and biochemical) 20% and 67%, respectively.

**Table 1:** Clinical, biochemical and ultrasonographic characteristics of PCOS patients

Variables	Mean ± SD or n (%)
Age (year)	29.92 ± 5.30
BMI (kg/m^2^)	28.57 ± 5.41
Waist circumference (cm)	96.59 ± 12.57
Menstrual cycle	
Regular	9 (14.80)
Irregular	52 (85.20)
Disease duration (year)	7.01 ± 6.36
Total testosterone (ng/mL)	0.46 ± 0.19
SHBG (nmol/L)	57.45 ± 49.29
FAI	5.08 ± 4.43
LH (mIU/mL)	14.11 ± 14.26
FSH (mIU/mL)	5.86 ± 2.55
LH/FSH ratio	2.30 ± 1.84
FBS (mg/dL)	88.90 ± 13.11
Insulin (µIU/mL)	13.23 ± 4.91
HOMA-IR	2.96 ± 1.38
TSH (mIU/mL)	2.53 ± 1.30
PCOM	
Unilateral	25 (41)
Bilateral	36 (59)
OV (cm^3^)	
Left	13.41 ± 4.76
Right	14.20 ± 4.77
^a^Right + Left	13.55 ± 4.26
^b^AFC	60.22 ± 29.78
^a^FNPO	
2–5 mm	30.10 ± 15.28
6–9 mm	4.03 ± 3.95
2–9 mm	37.02 ± 17.45
^a^Ovarian area (cm^2^)	7.41 ± 2.12
^a^Stromal area (cm^2^)	3.04 ± 2.05
^a^S/A ratio	0.42 ± 0.16

*AFC* Antral follicle count, *BMI Body mass index,* *FAI* Free androgen index, *FBS* Fasting blood sugar, *FNPO* Follicle number per ovary, *FSH* *Follicle-stimulating hormone*, *HOMA-IR* Homeostasis model assessment of insulin resistance*,* *LH* *Luteinizing hormone*, *OV* Ovarian volume, *PCOM* Polycystic ovarian morphology, *S/A ratio* Stromal to ovarian area ratio, *SHBG Sex hormone-binding globulin*, *TSH* Thyroid stimulating hormone

^a^The mean value of the left and right ovary was used for analysis

^b^Sum of follicles in both ovaries

The associations between testosterone, FAI, LH/FSH ratio and HOMA-IR, and ultrasonographic parameters are shown in Table 2. According to our results, bilateral PCOM had a significant relationship with a high testosterone level and a high FAI independent of age, menstruation status and BMI. However, no relationship was found between HOMA-IR, LH/ FSH ratio and bilateral PCOM (Table 2).

**Table 2:** Association of ovarian volume and morphology with hormonal parameters

Variables	Testosterone		FAI		LH/FSH		HOMA-IR	
	β (SE)	*P* value	β (SE)	*P* value	β (SE)	*P* value	β (SE)	*P* value
**PCOM (bilateral vs. unilateral)**								
Model 1 (unadjusted)	0.098 (0.051)	0.060	0.285 (0.099)	0.006	-0.162 (0.090)	0.079	-0.025 (0.052)	0.629
Model 2	0.104 (0.051)	0.044	0.289 (0.100)	0.005	-0.160 (0.091)	0.086	-0.028 (0.052)	0.597
Model 3	0.116 (0.052)	0.028	0.252 (0.101)	0.015	-0.166 (0.094)	0.084	-0.044 (0.053)	0.402
Model 4	0.119 (0.052)	0.028	0.216 (0.096)	0.029	-0.153 (0.095)	0.113	-0.068 (0.048)	0.164
**Right OV (cm**^**3**^**)**								
Model 1 (unadjusted)	0.020 (0.005)	< 0.001	0.037 (0.010)	0.001	0.027 (0.009)	0.006	0.001 (0.006)	0.945
Model 2	0.020 (0.005)	< 0.001	0.037 (0.010)	0.001	0.027 (0.009)	0.007	0.001 (0.006)	0.935
Model 3	0.020 (0.005)	< 0.001	0.038 (0.010)	< 0.001	0.027 (0.010)	0.008	0.001 (0.006)	0.899
Model 4	0.021 (0.005)	< 0.001	0.033 (0.010)	0.001	0.030 (0.009)	0.002	-0.003 (0.005)	0.573
**Left OV (cm**^**3**^**)**								
Model 1 (unadjusted)	0.021 (0.006)	0.001	0.041 (0.011)	< 0.001	0.015 (0.011)	0.166	0.001 (0.006)	0.952
Model 2	0.020 (0.006)	0.001	0.040 (0.011)	0.001	0.014 (0.011)	0.192	0.001 (0.006)	0.959
Model 3	0.020 (0.006)	0.002	0.044 (0.011)	< 0.001	0.016 (0.011)	0.152	0.001 (0.006)	0.892
Model 4	0.024 (0.006)	0.001	0.037 (0.012)	0.003	0.024 (0.012)	0.047	-0.007 (0.006)	0.243
^**a**^**Right + Left OV (cm**^**3**^**)**								
Model 1 (unadjusted)	0.022 (0.005)	< 0.001	0.045 (0.011)	< 0.001	0.025 (0.010)	0.017	0.001 (0.006)	0.947
Model 2	0.022 (0.005)	< 0.001	0.045 (0.011)	< 0.001	0.025 (0.010)	0.018	0.001 (0.006)	0.955
Model 3	0.022 (0.005)	< 0.001	0.047 (0.010)	< 0.001	0.025 (0.010)	0.019	0.001 (0.006)	0.964
Model 4	0.025 (0.006)	< 0.001	0.041 (0.011)	< 0.001	0.032 (0.011)	0.004	-0.006 (0.006)	0.288
^**b**^**AFC**								
Model 1 (unadjusted)	0.001 (0.001)	0.204	0.003 (0.002)	0.048	-0.001 (0.002)	0.669	0.002 (0.001)	0.046
Model 2	0.001 (0.001)	0.186	0.003 (0.002)	0.051	-0.001 (0.002)	0.685	0.002 (0.001)	0.049
Model 3	0.001 (0.001)	0.119	0.003 (0.002)	0.110	-0.001 (0.002)	0.658	0.001 (0.001)	0.103
Model 4	0.001 (0.001)	0.122	0.001 (0.002)	0.383	0.001 (0.002)	0.858	0.001 (0.001)	0.346
^**a**^**2-5 mm FNPO**								
Model 1 (unadjusted)	0.002 (0.002)	0.389	0.006 (0.004)	0.151	0.004 (0.004)	0.303	0.004 (0.002)	0.029
Model 2	0.001 (0.002)	0.529	0.006 (0.004)	0.175	0.003 (0.004)	0.417	0.004 (0.002)	0.020
Model 3	0.002 (0.002)	0.393	0.006 (0.004)	0.183	0.003 (0.004)	0.453	0.004 (0.002)	0.030
Model 4	0.002 (0.002)	0.417	0.003 (0.004)	0.487	0.004 (0.004)	0.317	0.003 (0.002)	0.088
^**a**^**6-9 mm FNPO**								
Model 1 (unadjusted)	0.003 (0.007)	0.678	-0.008 (0.014)	0.564	0.001 (0.013)	0.989	0.002 (0.007)	0.751
Model 2	0.006 (0.007)	0.380	-0.007 (0.014)	0.613	0.004 (0.013)	0.788	0.001 (0.007)	0.851
Model 3	0.007 (0.007)	0.379	-0.007 (0.014)	0.596	0.004 (0.013)	0.788	0.001 (0.007)	0.862
Model 4	0.007 (0.007)	0.378	-0.006 (0.012)	0.603	0.003 (0.013)	0.803	0.002 (0.006)	0.807
^**a**^**2-9 mm FNPO**								
Model 1 (unadjusted)	0.001 (0.001)	0.331	0.003 (0.003)	0.299	0.003 (0.003)	0.276	0.003 (0.001)	0.021
Model 2	0.002 (0.001)	0.283	0.003 (0.003)	0.309	0.003 (0.003)	0.260	0.003 (0.001)	0.024
Model 3	0.002 (0.001)	0.226	0.002 (0.003)	0.440	0.003 (0.003)	0.267	0.003 (0.001)	0.041
Model 4	0.002 (0.002)	0.233	0.001 (0.003)	0.899	0.004 (0.003)	0.142	0.002 (0.001)	0.184
^**a**^**Ovarian area (cm**^**2**^**)**								
Model 1 (unadjusted)	0.017 (0.014)	0.240	0.031 (0.027)	0.248	0.029 (0.024)	0.245	0.014 (0.013)	0.276
Model 2	0.014 (0.014)	0.321	0.030 (0.027)	0.269	0.028 (0.025)	0.270	0.014 (0.013)	0.289
Model 3	0.014 (0.014)	0.345	0.032 (0.027)	0.236	0.028 (0.025)	0.268	0.015 (0.013)	0.250
Model 4	0.013 (0.014)	0.364	0.027(0.025)	0.227	0.029 (0.025)	0.259	0.013 (0.012)	0.305
^**a**^**Stromal area (cm2)**								
Model 1 (unadjusted)	0.009 (0.013)	0.502	0.031 (0.025)	0.214	0.008 (0.023)	0.726	0.013 (0.012)	0.275
Model 2	0.007 (0.013)	0.607	0.032 (0.025)	0.204	0.007 (0.024)	0.777	0.014 (0.012)	0.251
Model 3	0.007 (0.013)	0.578	0.030 (0.025)	0.229	0.007 (0.024)	0.784	0.013 (0.012)	0.280
Model 4	0.007 (0.013)	0.572	0.035 (0.022)	0.121	0.005 (0.024)	0.835	0.016 (0.011)	0.165
^**a**^**S/A**								
Model 1 (unadjusted)	0.005 (0.190)	0.978	-0.102 (0.354)	0.775	0.169 (0.322)	0.602	0.085 (0.174)	0.629
Model 2	-0.021 (0.188)	0.912	-0.114 (0.359)	0.752	0.158 (0.326)	0.631	0.082 (0.176)	0.643
Model 3	-0.011 (0.188)	0.955	-0.140 (0.356)	0.697	0.153 (0.330)	0.646	0.069 (0.174)	0.694
Model 4	0.005 (0.195)	0.978	0.083 (0.337)	0.806	0.131 (0.343)	0.703	0.184 (0.163)	0.264

*AFC* antral follicle count, *FAI* free androgen index, *FNPO* follicle number per ovary, *FSH* follicle-stimulating hormone, *HOMA-IR* homeostasis model assessment of insulin resistance, *LH* luteinizing hormone, *OV* ovarian volume, *PCOM* polycystic ovarian morphology, *S/A* stromal to ovarian area ratio

Model 2 includes variables from model 1 plus age; Model 3 includes variables from model 1 plus age and mensuration status; Model 4 includes variables from model 1 plus age, mensuration status and BMI

^a^The mean value of the left and right ovary was used for analysis

^b^Sum of follicles in both ovaries

The right and left ovarian volume was positively related to the testosterone level, FAI and a high LH/FSH ratio in PCOS women after adjustments for age, menstruation status and BMI. When the mean volume of both ovaries was considered, the calculated mean volume was also significantly associated with a high testosterone level (β = 0.025, *P* value < 0.001), a high FAI (β = 0.041, *P* value < 0.001) and a high LH/FSH ratio (β = 0.032, *P* value = 0.004), even after full adjustments (Table 2). Ovarian volume (i.e. left, right and mean value of both ovaries) was not, however, associated with HOMA-IR before and after adjustments for age, menstruation status and BMI (Table 2).

With the exception of the significant association between FAI and AFC in the unadjusted model, AFC was not associated with androgen levels and FSH/LH ratio. Moreover, after adjustments with menstruation status and/or BMI, the significant relationship between AFC and insulin resistance was also cancelled out (Table 2).

No significant association was observed between 2 and 5 mm FNPO, 6–9 mm FNPO and 2–9 mm FNPO, and the testosterone level, FAI and LH/FSH ratio before and after adjustments for age, menstruation status and BMI. In the unadjusted model, 2–5 mm FNPO and 2–9 mm FNPO were associated with HOMA-IR. These significant associations were maintained after adjustments for age and menstruation status. However, after including BMI in the adjustment model, the significant association between FNPO and HOMA-IR was disappeared. We found no association between ovarian area, stromal area and S/A ratio, with androgens levels, LH/FSH ratio and HOMA-IR, regardless of the adjustment models (Table 2).

## Discussion

This study was conducted to assess the hypothesis that some aspects of the ovarian morphology using TVUS would reflect the degree of hormonal and metabolic disturbance in women with PCOS. Our results showed that bilateral PCOM had the highest predictive performance in identifying higher levels of testosterone and FAI in the patients with PCOS, thus indicating that the bilateral PCOM could also be more aggressive, as compared with the unilateral once. In addition, among the number of diagnostic variables in PCOM, ovarian volume could be the best parameter to distinguish PCOS women with hormonal imbalance from the normal ones; we showed that ovarian size was positively associated with total testosterone, FAI and LH/FSH ratio, whereas associations with HOMA-IR did not reach statistical significance. In other words, ovarian volume was an independent predictor of the elevated levels of hormones in PCOS, which was consistent with the previous findings [17, 18]. This is, thus, in agreement with the hypothesis that the excess androgen production, resulting from hypertrophic theca cells, is involved in the greater ovarian volume of the affected women [19].

In another study, Rackow et al., using stepwise linear regression analyses, concluded that total testosterone was related to ovarian volume, ovarian area, and follicle number in PCOS [20]. The differences between our results and those of Rackow et al. (ovarian volume is only related to androgen levels in our study) could be because their study was limited to 33 adolescents (12–18 years) with PCOS and used transabdominal ultrasound [20]. Given the better detection rate of smaller follicles (e.g., 3–4 mm) and higher accuracy in counting the number of follicles by the vaginal route [21], it may not be appropriate to make direct comparisons between the data provided by different imaging technologies (transabdominal in the study done by Rackow et al., as compared to TUVS applied in our study) and different age groups (the age of 12–18 in the study by Rackow et al., as compared to that of 18–40 in our study).

In accordance with the previous reports [20, 22], our results indicated that increased ovarian volume could also serve as a biomarker of the raised LH/FSH ratio in the PCOS women. In the ovary, androgens are produced by theca cells in response to LH stimulation [23]; defects of gonadotropins secretion, including elevated levels of LH or an elevated LH/FSH ratio, are the key factors associated with the persistence of the anovulatory cycles in the PCOS patients [22]. Additionally, it has been shown that the measurement of the serum LH level and the LH/FSH ratio may reflect the ovarian volume better than the serum AMH level in the PCOS subjects [22].

Excess 2–9 mm antral follicle number, a marker of follicle arrest, is known to be an oligo-anovulation risk factor [24] that can predict hyperandrogenism [25] and PCOS [26]. However, based on our results, the number of 2–9 mm follicles, as evaluated by AFC or FNPO, could not predict hormonal (total testosterone, FAI and LH/FSH ratio) or metabolic disturbance (HOMA-IR). Even after a subanalysis of the FNPO parameter with the use of different follicle sizes (2–5 or 6–9 mm), we did not find any association between follicle count and testosterone, FAI, LH/FSH ratio, or HOMA-IR. Our findings were, thus, in contrast with the previous works which focused on the follicle numbers in the range of 2–5 mm and 6–9 mm; for example, Brink et al. observed that the number of follicles with the size of 6–9 mm was predictive of total testosterone in healthy women with normal menstrual cycles, but not among those with irregular menstrual cycles [6]; meanwhile, Jonard et al. found that this relationship was limited to the number of follicles with the range of 2–5 mm [27]. These conflicting findings could be due to differences in the techniques used for measuring testosterone levels [6], population of study [6] and sample size [27].

In our study, we also found a null association between stromal area, ovarian area and S/A, and abnormal androgen levels. It is hard to find studies evaluating such parameters in relation to hyperandrogenism in literature [18]. However, in a study done by Fulghesu et al., S/A, stromal area and ovarian volume were also associated with higher androgen levels. They also proposed a cut off of 0.32 for the upper limit value of S/A for both androstenedione and testosterone [16]. The sample size of Fulghesu et al. study was calculated based on the S/A using the 0.90 power and the level of significance of 0.05 (n = 280). That we did not find an association between S/A and androgens may be because our sample size was not large enough to detect associations between androgens and more detailed outcomes such as S/A. The use of stromal volume for the diagnosis of hyperandrogenemia is hindered by the fact that stromal volume and ovarian size are well correlated; so, adding it to the general routine practice does not provide much value [28].

We also found no associations between ovarian ultrasound parameters and insulin resistance. Similarly, a study which assessed the morphology and size of the ovaries in women with PCOS by ultrasound demonstrated that neither the morphology nor the size of the ovaries was associated with any parameter of insulin action [29]. They used more accurate and dynamic assessments of insulin sensitivity, including the measurement of insulin and glucose concentrations in the fasting (0 min) and after 120-min oral glucose tolerance test (OGTT) [29]. Our study used HOMA-IR, reaching a similar conclusion. However, while other studies found a positive association between ovarian volumes and insulin resistance in women with PCOS [8, 30], these studies suffered from low sample size [31] or modest association [32]. In addition, Hong et al. [9] demonstrated that FNPO best predicted insulin resistance in PCOS. A potential explanation for these discrepancies between studies might be related to the differences in the criteria used to diagnose patients with PCOS and insulin resistance.

Finally, with the advancement of ultrasound equipment, TVUS is becoming an excellent modality for the high resolution imaging of ovaries. The diagnosis performance of TVUS in assessing both ovarian volume and follicle counting in PCOS is generally higher than that of transabdominal examination. Furthermore, abdominal ultrasonography is not suitable for detecting the exact number of antral follicles, and it can be difficult to count antral follicles by the excess abdominal fat [33]. However, despite the superiority of assessing follicle count, TUVS is far from an ideal gold standard because it requires a probe with frequency ≥ 7 MHz, which is less available and needs a longer scanning time [34]; doctors rarely advocate it, and patients (virgin or refusing patients) hardly select it when given the option. Our finding, thus, adds to the current evidence that the ovarian volume measurement is sufficient to meet the diagnosis of biochemical androgen excess. Ovarian volume is an attractive surrogate for hyperandrogenism in many ways as it is easy to obtain with abdominal sonography.

The strengths of our study include the use of TVUS for the accurate diagnosis of PCOM, the use of Rotterdam criteria to define POCS and the use of a relatively homogeneous population. The present study also faced some limitations. We included only a small number of patients and there was a lack of comparison with the control group. The sample size of our study specifically for S/A and HOMA-IR may not be sufficiently powered to identify an association with PCOM. Lack of a control group makes it difficult to determine whether the relationships we found have a similar magnitude when compared to women without PCOS.

However, to date, there is a paucity of published literature regarding the predictive role of PCOM for women with PCOS. At present, PCOM employed in PCOS is based mostly on the Rotterdam study; thus, continued study is still needed in this direction. Furthermore, our research was based on a cross-sectional design; therefore, causality cannot be established. In addition, recent studies indicate that ultrasound cut-offs for the diagnosis of PCOS may vary according to ethnicity; for example, FNPO is much lower in Asian Indian women, in comparison to their Caucasian counterparts [35]; so, application of these results to other populations is limited. Finally, Liquid chromatography–mass spectrometry (LC/MS) is currently the preferred technique for measuring testosterone due to its higher accuracy. However, the method we used to measure testosterone was ELISA, which can affect our results.

## Conclusion

Increased ovarian volume is highly predictive of hyperandrogenemia in PCOS patients and strongly associated with the serum total testosterone level, LH/FSH ratio and FAI index. Ovarian volume represents the best ultrasound marker of PCOS; more likely than follicle count, it has an outstanding position in the diagnosis of the excess androgen levels of PCOS patients and more prudent to measure the ovarian volume alone because of FNPO dependence on the high quality image and the increased time to evaluate both ovarian volume and FNPO.

## Data Availability

Data are available upon reasonable request. To access the required data, the researchers can contact Dr. Asieh Mansour, Email:asiehmansour@yahoo.com.
